# Band Structure Near the Dirac Point in HgTe Quantum Wells with Critical Thickness

**DOI:** 10.3390/nano12142492

**Published:** 2022-07-20

**Authors:** Alexey Shuvaev, Vlad Dziom, Jan Gospodarič, Elena G. Novik, Alena A. Dobretsova, Nikolay N. Mikhailov, Ze Don Kvon, Andrei Pimenov

**Affiliations:** 1 Institute of Solid State Physics, Vienna University of Technology, 1040 Vienna, Austria; shuvaev@ifp.tuwien.ac.at (A.S.); jan.gospodaric@tuwien.ac.at (J.G.); 2 Institute of Science and Technology Austria, 3400 Klosterneuburg, Austria; uladzislau.dziom@ist.ac.at; 3 Institute of Theoretical Physics, Technische Universität Dresden, 01062 Dresden, Germany; alena.astakhova@tu-dresden.de; 4 Rzhanov Institute of Semiconductor Physics, 630090 Novosibirsk, Russia; dobretsovaaa@gmail.com (A.A.D.); mikhailov@isp.nsc.ru (N.N.M.); kvon@isp.nsc.ru (Z.D.K.); 5 Department of Physics, Novosibirsk State University, 630090 Novosibirsk, Russia

**Keywords:** Dirac fermions, topological insulators, band structure, quantum wells, cyclotron resonance

## Abstract

Mercury telluride (HgTe) thin films with a critical thickness of 6.5 nm are predicted to possess a gapless Dirac-like band structure. We report a comprehensive study on gated and optically doped samples by magnetooptical spectroscopy in the THz range. The quasi-classical analysis of the cyclotron resonance allowed the mapping of the band dispersion of Dirac charge carriers in a broad range of electron and hole doping. A smooth transition through the charge neutrality point between Dirac holes and electrons was observed. An additional peak coming from a second type of holes with an almost density-independent mass of around $0.04m_{0}$ was detected in the hole-doping range and attributed to an asymmetric spin splitting of the Dirac cone. Spectroscopic evidence for disorder-induced band energy fluctuations could not be detected in present cyclotron resonance experiments.

## 1. Introduction

Electronic band structure in mercury telluride (HgTe) quantum wells (QW) can be engineered through the growth procedure [1]. For HgTe QWs with the thickness below critical $d_{c}≃6.5$ nm the energy gap opens between H1 valence and E1 conduction bands and an insulating state is realized when the Fermi energy lies within the gap. Both bands are touching at $d=d_{c}$ forming a two-dimensional (2D) semimetal with zero gap and with Dirac dispersion [2]. Further increasing of the thickness leads again to an opening of a gap and to an appearance of topological edge states resulting in a quantum spin Hall insulator [1,2,3]. If a bulk HgTe layer d≳50 nm is grown on a CdTe substrate, tensile strain due to lattice mismatch splits the originally degenerate light and heavy $\Gamma_{8}$ hole bands at the center of the Brillouin zone, forming a three-dimensional topological insulator [4,5] with a surface state that connects valence and conduction bands [6]. In addition, quantum wells based on cadmium-mercury tellurides (HgTe/Cd${}_{1-x}$Hg${}_{x}$Te) can be used as emitters and detectors of the far-infrared radiation [7,8]. The physics here is similar to that of quantum well-based light-emitting diodes for visible light [9].

HgTe wells in the thickness range of about 10–30 nm represent a two-dimensional semimetal, where electrons and holes coexist simultaneously [10,11]. Here the H2 valence and H1 conduction bands indirectly overlap forming a negative gap in the meV range [12]. On the contrary, HgTe wells with thickness around $d_{c}≃6.5$ nm demonstrate vanishing gap in transport measurements. The interest in these quantum wells was enhanced substantially when the calculation of the band structure using k·p model [2] predicted a topological transition with the relativistic electron spectrum $E\left(k\right)=v_{F}\hbar k$ around critical thickness.

Although the electronic band structure provides an important fingerprint of a material in the reciprocal space, in the case of heterostructures, the standard technique of angle-resolved photoemission (ARPES) [13] cannot be applied due to multiple layers. To recover the band structure of HgTe films, an alternative procedure was suggested that uses an analysis of the cyclotron resonance in the terahertz frequency range, while the Fermi level in the films is being shifted by external parameters [6,12,14]. Due to the quasi-classical regime of the cyclotron resonance, the results can be well processed via the standard electrodynamic approach. Such an approach provides sufficient information about the carriers in the system, enabling direct access to the band dispersion.

Continuous improvement in the growth technology [15] and a creation of semi-transparent gates made it possible to use magneto-optical terahertz (THz) spectroscopy to study the electronic spectrum around the Dirac point. In the present work, we report the band structure of HgTe quantum wells with critical thickness reconstructed from systematic study of the cyclotron resonance. Our experiments provide direct evidence of significant electron-hole asymmetry around the Dirac point, which we attribute to much stronger spin splitting in the valence band. Particle-hole asymmetry, which can be useful in quantum devices such as nano-transistors [16] and magnetically tunable lasers [17], has been long overlooked in theoretical models for CdHgTe heterostructures. The technique used in this work can be successfully applied to other 2D materials such as graphene [18], transition metal dichalcogenides [19], or CdSe [20].

## 2. Experimental Section

### 2.1. HgTe Quantum Wells

Mercury telluride thin films with critical thickness of 6.5 nm were grown by molecular beam epitaxy on (013)-oriented GaAs substrates. The quantum wells are stacked between two 30 nm Cd${}_{0.65}$Hg${}_{0.35}$Te layers to increase the charge carrier’s mobility. The symmetrical doping by Indium was introduced in the middle of these buffer and capping layers to put the Fermi level of the system close to charge neutrality point. A semitransparent gate was fabricated at sample #1. It consists of 400 nm multilayered SiO${}_{2}$/Si${}_{3}$N${}_{4}$ insulator and 10.5 nm Ti-Au gate electrode. The shape of the gate electrode provides a space for a 4 mm optical aperture in the sample center and for four ohmic contacts in the corners. We note that the remaining regions of the ungated film at the sample edges will shunt the gated area and falsify the transport data at nonzero gate voltages. Therefore, direct comparison of the THz data with static results was possible at zero gate voltage only. The sample #2 without gate allowed to use 10 mm aperture and possessed four ohmic contacts in the corners. A modification of the charge density in this sample is achieved through illumination with green LED using the effect of persistent photoconductivity [1,21,22,23,24].

### 2.2. Magneto-Optical Technique

Magneto-optical experiments in the THz frequency range are carried out using a quasi-optical technique [24,25]. Continuous radiation is produced by backward wave oscillators (BWOs). The polarization of the beam is controlled by wire grid polarizers. The sample is placed in the helium-cooled cryostat with the superconducting magnet and transparent windows made of mylar film. The main measurement mode in the present work is to fix the frequency of the incident radiation and to measure the transmitted intensity of the circularly polarized wave. The circular polarization is produced using an adjustable quarter-wavelength apparatus. The transmitted signal is measured as a function of an applied magnetic field and of the gate voltage.

## 3. Modeling

### 3.1. Optical Drude Conductivity

In the present case of an isotropic dielectric substrate and gyrotropic two-dimensional (2D) electron system in a perpendicular magnetic field, the electromagnetic eigenmodes are circularly polarized waves. They are directly accessible experimentally and it is thus natural to consider the transmittance coefficients in the circular basis. The total dimensionless circular conductivity $g_{\pm }=\sigma_{\pm }Z_{0}$ can be expressed as:

(1) $$g_{\pm }=g_{xx}\pm ıg_{yy}=\frac{g_{0}}{1-ı\tau (\omega \pm \Omega_{c})}.$$

here, $\Omega_{c}=eB/\left(m_{c}\right)$ is the cyclotron frequency, τ is the transport relaxation time, $g_{0}=\sigma_{0}Z_{0}$ is the dimensionless 2D DC conductivity, and $Z_{0}$ is the impedance of vacuum. In the case of multiple charge carriers their partial conductivities sum up. To take the effects of the substrate into account we define two substrate coefficients $s_{1}$ and $s_{2}$:

(2) $$\begin{array}{cc} s_{1} & =\cos(kd)-ıZ\sin(kd)\;,\\ s_{2} & =\cos(kd)-ı/Z\sin(kd)\;, \end{array}$$

where $k=\sqrt{\varepsilon }\omega /c$ is the wavevector of the THz wave in the substrate of thickness *d* and $Z=1/\sqrt{\varepsilon }$ is the normalized wave impedance of the substrate material. The complex transmission coefficient of circularly polarized wave is given by:

(3) $$t_{\pm }=\frac{2}{\left(1+g_{\pm }\right)s_{1}+s_{2}}.$$

The light intensity measured directly in the experiment is $I=|t_{\pm }{|}^{2}$. After fitting the experimental curves with Equations (1)–(3) the Drude parameters of the charge carriers $\sigma_{0}=ne^{2}\tau /m$, τ and $m_{c}$ can be easily obtained. Here we assume that the transport mass equals the cyclotron mass, $m=m_{c}$.

### 3.2. Reconstructing the Band Structure

The Fermi wavevector can be calculated in the isotropic approximation from the density of the charge carriers. For the two-dimensional case $k_{F}=\sqrt{4\pi n/D}$, where *D* is the degeneracy of states. The quasi-classical expression for the cyclotron mass

(4) $$m_{c}=\frac{\hbar^{2}}{2\pi }\frac{\partial A}{\partial E}{|}_{E=E_{F}}$$

is used to obtain the band dispersion within the isotropic model [6]:

(5) $$\frac{dE}{dk}{|}_{E=E_{F}}=\frac{\hbar^{2}k_{F}}{m_{c}}.$$

here *A* is the Fermi surface area and $k_{F}$ is the Fermi vector.

We note however that the isotropic approximation can be avoided by directly plotting $m_{c}\;vs.\;n$, see Figure 3 below.

### 3.3. **k**·**p** Model

Theoretical calculations were made using a multiband **k**·**p** model [26] which takes into account the coupling between the lowest conduction and the highest valence bands. Eight bands are taken into account in the present version of the model: two $\Gamma_{6}$, two $\Gamma_{7}$, and four $\Gamma_{8}$ subbands. The calculations include the structure inversion asymmetry and, therefore, they effectively reproduce the effect of the gate voltage. A similar procedure involves an interface inversion asymmetry [27]; however, it does not allow the calculation of the Hartree potential. Both approaches are qualitatively similar, they lead to the lifting of the spin-degeneracy of the bands. The Hartree approximation allows us to model the influence of an asymmetric gate potential at nonzero applied voltages.

To account for the inversion asymmetry terms in the model, structure and bulk inversion asymmetries [28] were taken into account. Within the model, variation in the doping in the top barrier is assumed, while the doping in the barrier on the substrate side is taken to be constant. Asymmetric barrier doping results in the asymmetric distribution of the Hartree potential which has been determined by solving self-consistently the eigenvalue problem [26]. Bulk inversion asymmetry of the zinc-blende crystal structure gives rise to the Dresselhaus spin–orbit interaction. In the present case, the bulk inversion asymmetry terms are linear in momentum for the valence bands, while the contribution from the coupling between the conduction and valence bands is quadratic [28].

To obtain the density dependence of the cyclotron mass within the present theory, the effect of the gate voltage was modeled by varying the total charge density in the system with the Fermi level reaching the valence and conduction subbands, respectively. For each value of the charge density the cyclotron mass was calculated using Equation (4) as a function of density within the corresponding bands.

Finally, theoretical calculations confirmed the approximate rotational symmetry of the band structure close to the Dirac point, thus justifying the use of Equation (5) to reconstruct the energy dispersion from the cyclotron data.

## 4. Results

### 4.1. Cyclotron Resonance

**Figure 1:**
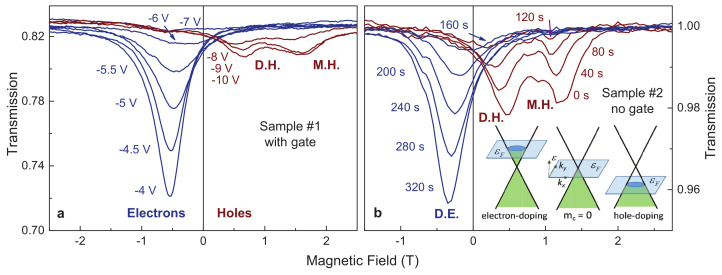
Magneto-transmission of a circularly polarized light in HgTe quantum wells with critical thickness at T=1.8 K and ν=950 GHz. (**a**) Sample #1 with gate (gate voltage is given at curves). (**b**) Sample #2 without gate. The charge density here is varied via light illumination with illumination time as indicated. Negative and positive magnetic fields correspond to negatively- and positively-charged quasiparticles, respectively. Acronyms: D.E.—Dirac electrons, D.H.—Dirac holes, M.H.—massive holes (see text for details). The inset in (**b**) demonstrates schematically the expected Fermi surface for different doping levels.

Representative magnetic field dependencies of the transmittance are shown in Figure 1. The high frequency of 950 GHz facilitates the observation of low carrier masses $m_{c}$ close to the Dirac point in these experiments. The experiments at other frequencies demonstrated similar results confirming the linear relation $\Omega_{c}\propto B_{c}$, as expected in the quasi-classical limit. The normalized transmission of the circular amplitude $|t_{+}|$ through two samples is shown either at different gate voltages (sample #1) or as function of illumination time (sample #2). The cyclotron resonance coming from the Dirac electrons (D. E.) is clearly visible at negative magnetic fields (blue curves in Figure 1). By approaching the charge neutrality point, the amplitude of the resonance decreases and the peak position shifts toward zero field. The presence of the resonance shift indicates strong non-parabolicity of the band structure. The simple Equation (5) predicts $m_{c}∼\sqrt{n}$ dependency for the linear Dirac dispersion. On the other side of the charge neutrality point, at gate voltages below −6.5 V (sample #1) or illumination time below 160 s, the two cyclotron resonance peaks appear at positive magnetic fields (brown curves in Figure 1). The peak with the position starting from zero magnetic fields is coming from Dirac holes (D. H.).

**Figure 2:**
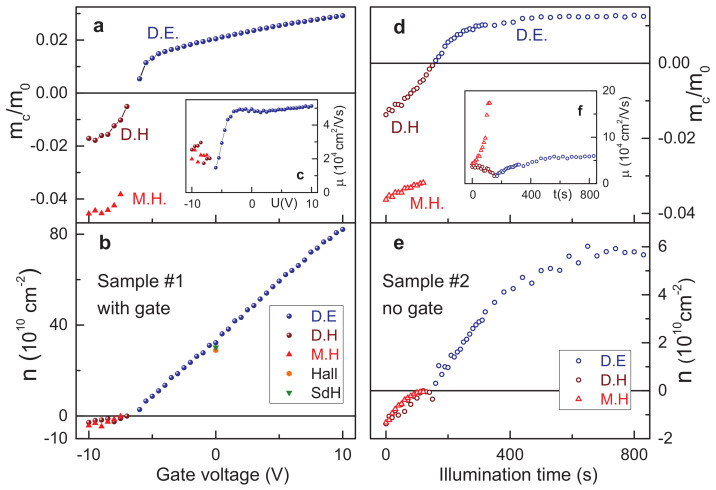
Parameters of the charge carriers as extracted from the Drude fits of the cyclotron resonance. (**a**–**c**) Sample #1 with gate as function of gate voltage, (**d**–**f**) Sample #2 without gate as function of illumination time. (**a**,**d**) Cyclotron mass, (**b**,**e**) Charge density, (**c**,**f**) Mobility. Acronyms: blue circles-Dirac electrons (D.E.), brown circles-Dirac holes (D.H.), red triangles-massive holes (M.H.).

Simultaneously, in the hole-doping range a second peak appears at finite fields, which is the signature of the charge carriers with nonzero cyclotron mass. Therefore, these charge carriers will be designated as massive holes (M. H.). The transition through the Dirac point is seen in better detail in the gate-free sample #2. Here, both the overall change and the step size of the density are smaller than on the gated sample #1. When cooled in the dark, sample #2 shows two hole-like cyclotron resonances. By illuminating the sample with the green LED the amplitude of these peaks decreases. The inset in Figure 1b demonstrates schematically the expected Fermi surface for different doping levels. From the spectra in Figure 1 the designation of Dirac charge carriers is obvious: it is the resonance peak that continuously passes through zero magnetic field (which corresponds to the cyclotron mass crossing zero as well, see Figure 2).

It can be expected that due to spatial deviations of the quantum-well thickness from the critical value of 6.5 nm, different parts of the sample will be phase-separated and thus slightly electron or hole doped [29]. These differently doped areas would result in simultaneous observation of cyclotron resonances from electrons and holes. Although the percolation hypothesis has been supported by static results [29], in the present dynamic experiments we do not see electron/hole splitting of the resonance. Instead, the transition between negative and positive resonance fields is continuous. Most probably, in contact-free optical experiments the sample properties are effectively averaged by the high frequency.

### 4.2. Drude Parameters

The parameters of the charge carriers can be extracted by fitting the experimental data in Figure 1 with the Drude model, Equations (1)–(3). The position of the cyclotron resonance gives the cyclotron mass $m_{c}$, the amplitude is proportional to the density of the carriers *n* and the width of the peak contains the information about the mobility μ. The obtained parameters are shown in Figure 2. The left panels a-c represent the parameters for the sample #1 as a function of gate voltage, the right panels d-f demonstrate the data for the sample #2 as a function of the cumulative illumination time.

The most noticeable result on both samples is the cyclotron mass of Dirac carriers continuously crossing zero as shown in panels a and d. This is an expected outcome for the charge carriers with a linear dispersion when the Fermi level crosses the Dirac point. Here, the mass is assumed to be positive for electrons and negative for holes. Other detected charge carriers such as massive holes show only weak density dependence of the cyclotron mass. Such behavior is a signature of the parabolic dispersion with slight distortions.

Panels b and e in Figure 2 show the density of the charge carriers in both samples. Here, the density is plotted as positive for electrons and negative for holes. Dirac electrons in the gated sample demonstrate a linear filling rate $dn/dU=5\times 10^{10}$ cm${}^{-2}$V${}^{-1}$. The value expected from the geometrical structure of the sample is closely similar: $dn/dU=\varepsilon \varepsilon_{0}/\left(ed\right)\approx 6.4\times 10^{10}$ cm${}^{-2}$V${}^{-1}$. As a consistency check, the electron densities obtained from the magnetotransport at zero gate voltage are shown in Figure 2b as well. The orange diamond is the density from the slope of the Hall resistivity $dR_{xy}/dB$ and the green triangle represents the value obtained from the periodicity of the Shubnikov-de Haas (SdH) oscillations. Both values agree well with the data from the fitting of cyclotron resonance.

The filling rate (D.H + M.H., Figure 2b) drops roughly by a factor of two on appearance of holes in the system. One possible reason could be that the Fermi level already interacts with the heavy-hole pockets [30,31]. Due to a higher mass, these states are characterized by low mobility and high density of states. Their influence can be expected around 10–20 meV below the Dirac point. The charge carriers with mobility that is too low do not produce a distinct cyclotron resonance and remain undetected in the experiment. Therefore, the filling rate obtained only from the observed carriers is reduced. The density dependence in the gate-free sample #2 is shown in Figure 2e. In this case, the influence of hole pockets on the charge density is not observed. The density rises almost linearly up to 400 s of illumination time and then saturates above this value.

Panels c and f in Figure 2 show the mobility of the charge carriers. Dirac electrons reveal the mobility around $5\times 10^{4}$ cm${}^{2}/$Vs at high densities in both samples. The mobility drops down at low densities in the vicinity of the Dirac point due to reduced screening of the random potential [32]. Dirac holes are more mobile in the sample without gate. The most unusual result is the noticeable increase of the mobility of the massive holes in this sample when the Fermi level approaches the Dirac point.

### 4.3. Electronic Band Structure

**Figure 3:**
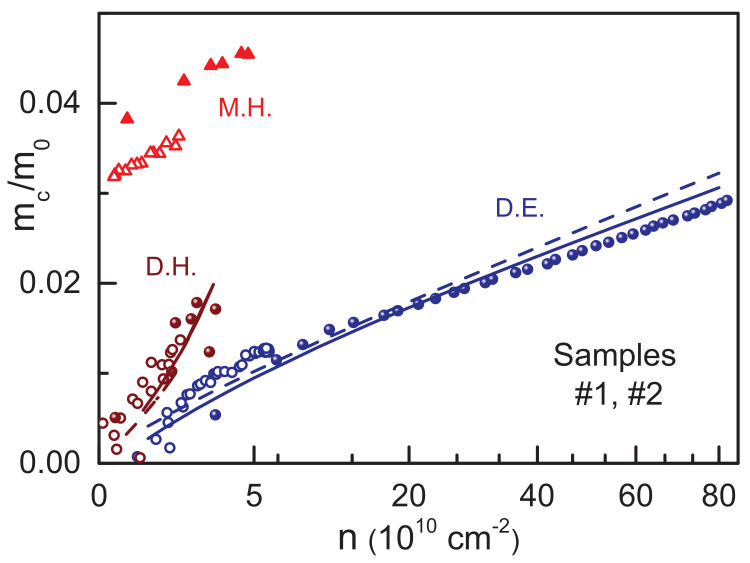
Cyclotron mass $m_{c}$ as a function of density *n*. Note the square root scale of the horizontal axis. The data from the gated sample #1 are shown by filled symbols, from the sample #2 without gate-by open symbols. Dirac electrons and holes are given by dark blue and brown circles, respectively. Massive holes are given by red triangles. The blue and brown lines are the predictions by the k·p model for Dirac electrons and holes, respectively. The difference between solid and dashed lines is due to splitting in the asymmetric potential and to the bulk inversion asymmetry.

Having both, the cyclotron mass and the density of the charge carriers, it is possible to plot $m_{c}$ as a function of *n*. The main advantage of such a representation is the ability to directly compare experimental data from the Drude fits with theoretical curves from the band structure calculations. Importantly, this presentation does not require isotropic approximation. This comparison is made in Figure 3. Both data from samples #1 and #2 are combined and are shown by filled and open symbols, respectively. The lines are calculated within the k·p model. The difference between solid and dashed lines is the manifestation of the lifting of the two-fold degeneracy due to an asymmetric potential within the film and to the bulk inversion asymmetry. Both electrons and holes are only weakly affected by this splitting and it can be ignored in the $m_{c}\left(n\right)$ representation in Figure 3.

We observe a good agreement between experiment and theory for Dirac carriers in Figure 3. Both masses show an approximate square root dependence $m_{c}∼\sqrt{n}$, typical for the linear Dirac-like dispersion [21,24,33,34,35]. Note that an approximate square root behavior is observed in a very broad range of densities from 0.3$\;\times \;10^{10}$ cm${}^{-2}$ to $80\times 10^{10}$ cm${}^{-2}$. Dirac holes are heavier than the Dirac electrons with the same density and their cyclotron mass grows rapidly at higher densities deviating from the square root law. This is the manifestation of the electron-hole asymmetry in the HgTe films with the critical thickness [36] and is due to the merging of the Dirac-like dispersion into the $\Gamma_{8}$ valence band with large effective mass.

Evidently, massive holes shown as red triangles in Figure 3 cannot be described by the present version of the model. Their cyclotron mass is around 0.04 of the free electron mass ($m_{0}$). It is too light to be explained by the side pockets of heavy holes with the cyclotron mass of around 0.2–0.4 of free electron mass [6,11,37].

**Figure 4:**
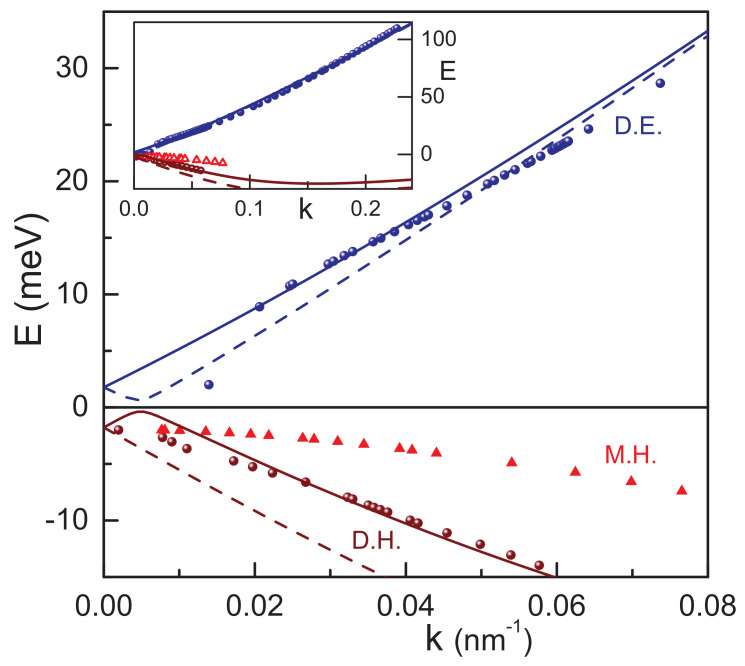
Band structure of HgTe films with critical thickness. Symbols are experimental data from two samples, lines—k·p theory. The symbol notations are the same as in Figure 3. The solid and dashed lines are split bands due to asymmetric potential and to bulk inversion asymmetry. The different slope of electrons and holes is due to a lifted hole degeneracy. The inset shows the overview over the data on a large energy scale. The flattening of the hole dispersion at high values of *k* is due to approaching of the hole pockets around k∼0.5 nm${}^{-1}$, E∼−15 meV.

Assuming that the Fermi surface is a circle at the center of the Brillouin zone, it is easy to find the Fermi wavevector $k_{F}=\sqrt{4\pi n/D}$, where *D* is the degeneracy of states. Only one electronic cyclotron resonance is seen above the Dirac point. It is thus natural to assume that the spin splitting of the Dirac electrons is too small and is not observed experimentally. Therefore, the degeneracy D=2 has to be used in this case. On the contrary, two different types of holes are observed below the Dirac point, and D=1 is appropriate. With the experimental values of wavevector *k* it is now possible to use Equation (5) to find the derivative dE/dk of the energy dispersion E(k). The band structure itself is recovered after numerical integration. Figure 4 shows the experimentally reconstructed bands (filled symbols) together with the theoretical calculations within the k·p model (lines). Already at this point it should be noted that the agreement between theory and experiment is good for Dirac electrons and holes. Massive holes are not well-described within the present calculations.

The theoretical curves shown in Figure 4 are calculated assuming a negative gate voltage corresponding to the hole density $p=1.4\times 10^{11}$ cm${}^{-2}$. This voltage together with the bulk inversion asymmetry (Section k·p *model*) is responsible for the band splitting visible in the plot. It is then possible to attribute the experimental Dirac holes (brown circles) to the lower theoretical split band (dashed line). We observe that the dispersion of the Dirac electrons is well-reproduced even for comparatively large wave vectors. This is especially remarkable as the model does not contain fitting variables. As mentioned above, the parameters of the model are the same as in Reference [26].

The massive holes (triangles) can be associated with the upper branch (solid line). Note that this upper branch has a local maximum at around k=0.005 nm${}^{-1}$ that would qualitatively lead to a density-independent cyclotron mass, at least in some narrow range. However, quantitative agreement with the present version of the model is not possible within a reasonable set of parameters. This probably indicates that further mechanisms of asymmetric spin splitting in the HgTe wells have to be taken into account. Several theoretical approaches to this problem were suggested recently [38,39]. In general, asymmetric terms break the inversion symmetry, thus leading to the spin splitting of the branches and to an appearance of an energy gap at the Dirac point. For example, the bulk inversion asymmetry provides a finite correction to the dispersion relations and even breaks the rotational symmetry of the quantum well. A typical value of this correction as well as that of the expected gap is ∼1 meV. Further theoretical and experimental efforts are necessary to resolve the splitting of the hole dispersion observed in this work.

## 5. Conclusions

Here we present the experimental and theoretical band structure of HgTe quantum wells with critical thickness that reveal linear dispersion near the Dirac cone. Experimental reconstruction of the band structure is obtained from the analysis of the cyclotron resonance at terahertz frequencies. This quasi-classical procedure is supported by the k·p model that confirmed the approximate rotational symmetry close to the Dirac point. At the electronic part of the band diagram a single cyclotron resonance is detected revealing a single electron-like dispersion branch. On the contrary, an additional signal of hole-like carriers with cyclotron mass of around $0.04\;m_{0}$ was detected and attributed to an asymmetric spin splitting of the Dirac cone.

A smooth transition through the charge neutrality point between Dirac holes and Dirac electrons was observed without signs of phase-separated electron-hole regions. In the latter case, we would expect simultaneous electron and hole cyclotron resonances in some range of doping. This is in contrast to static transport-based evidence for band structure instability due to thickness fluctuations. This difference might be explained by an effective averaging of the responses from electron and hole islands at terahertz frequencies.

## Data Availability

Data are available from the authors on reasonable request.
